# Family planning services quality as a determinant of use of IUD in Egypt

**DOI:** 10.1186/1472-6963-6-79

**Published:** 2006-06-22

**Authors:** Rathavuth Hong, Livia Montana, Vinod Mishra

**Affiliations:** 1 Demographic and Health Research Division, ORC Macro, 11785 Beltsville Drive, Calverton, MD 20705, USA; 2 George Washington University, School of Public Health and Health Services, 2300 Eye Street NW, Washington, DC 20037, USA

## Abstract

**Background:**

Both availability and quality of family planning services are believed to have contributed to increasing contraceptive use and declining fertility rates in developing countries. Yet, there is limited empirical evidence to show the relationship between the quality of family planning services and the population based prevalence of contraceptive methods. This study examined the relationship between quality of family planning services and use of intrauterine devices (IUD) in Egypt.

**Methods:**

The analysis used data from the 2003 Egypt Interim Demographic and Health Survey (EIDHS) that included 8,445 married women aged 15–49, and the 2002 Egypt Service Provision Assessment (ESPA) survey that included 602 facilities offering family planning services. The EIDHS collected latitude and longitude coordinates of all sampled clusters, and the ESPA collected these coordinates for all sampled facilities. Using Geographic Information System (GIS) methods, individual women were linked to a facility located within 10 km of their community. A facility-level index was constructed to reflect the quality of family planning services. Four dimensions of quality of care were examined: counseling, examination room, supply of contraceptive methods, and management. Effects of quality of family planning services on the use of IUD and other contraceptive methods were estimated using multinomial logistic regression. Results are presented as relative risk ratios (RRR) with significance levels (*p*-values).

**Results:**

IUD use among women who obtained their method from public sources was significantly positively associated with quality of family planning services (RRR = 1.36, *p *< 0.01), independent of distance to the facility, facility type, age, number of living children, education level, household wealth status, and residence. Quality of services related to counseling and examination room had strong positive effects on use of IUD (RRR = 1.61 for counseling and RRR = 1.46 for examination room). Obtaining IUD from a private source or using other contraceptive methods was not associated with quality of services.

**Conclusion:**

This study is one among the few that used geographic information to link data from a population-based survey with an independently sampled health facility survey. The findings demonstrate that service quality is an important determinant of use of clinical contraceptive methods in Egypt. Improving quality of family planning services may help further increase use of clinical contraceptive methods and reduce fertility.

## Background

Family planning and reproductive health programs have contributed greatly to fertility decline in developing countries [1]. Both availability and quality of family planning services are believed to have contributed to increasing contraceptive use and declining fertility rates in developing countries. There is general agreement that the quality of family planning and reproductive health services positively affects contraceptive use and behavior of the clients; and that clients deserve to receive safe and high quality services with respect and dignity [2].

Quality of care in family planning is a complex, multi-dimensional subject. For example, the Bruce framework of quality includes six quality indicators: choice of methods, information given to clients, technical competence, client-provider interpersonal relations, mechanisms to ensure follow-up and continuity, and the appropriate constellation of services [3]. International Planned Parenthood Federation's framework of "Client's Rights and Provider's Needs" includes client's rights to information, access to services, informed choice, safe services, privacy and confidentiality, dignity, comfort, and expression of opinion, and continuation of care; and provider's needs of facilitative supervision and management, information, training and development, and supplies, equipment and infrastructure [4]. Various indicators used to measure quality of care can be grouped into infrastructure and system readiness, provider's adherence to standards of practices, and client's perspectives and experiences. These dimensions of quality of care are interrelated. For example, provider's adherence to good practices is enhanced by training and supervision, and client's opinion about quality of services reflects provider practices.

There is limited empirical research to show the relationship between quality of family planning services and the population-based prevalence of contraceptive use, especially for the methods required to be administered at a health facility, and by a trained healthcare provider. Previous studies have assessed the effects of one or a few quality indictors of family planning services on the contraceptive use. One recent study measured quality of family planning services based on clients' reports of provider-client interactions; and examined its relationship with continuation of contraceptive methods at clients' follow-up visit in two provinces in the Philippines [2]. Another study in rural Bangladesh, defined quality based on clients' perception of practices and behaviors of family planning providers and associated it with their subsequent use of contraceptive methods [5]. Other family planning services quality indicators, such as availability of infrastructure, have also been shown to have positive effects on the use of contraceptive methods among new and returning clients [6,7]. Availability of visual aids supports providers in demonstrating and educating the clients about the methods and availability of guidelines reinforces provider's knowledge in administering the methods, especially for providers working independently in small, remote facilities. Other previous studies have indicated that availability of a broader range of contraceptive methods increases utilization of contraceptives through expanding method choice [8-10]. These studies, however, measured quality from different perspectives that represent only some aspects of quality of the family planning services.

Several other indicators are associated with the quality of family planning services. These include providers' adherence to standard practices, such as discussing issues and side effects of contraceptive methods and reproductive and medical history, as well as conducting basic examinations to ensure methods are administered safely, respecting clients' privacy, and tailoring counseling to meet clients' needs [11,12]. The quality of service can be enhanced by using other means of education such as group counseling and visual aids such as posters, leaflets, and videos [13,14]. The quality of services can also be improved by upgrading skills of the healthcare providers through in-service training and by providing personal supervision to the healthcare providers.

The type of contraceptive method used is associated with the source where the method is acquired. Clinical methods such as intrauterine devices (IUDs) and sterilization are generally administered at the healthcare facility where there are necessary equipment, supplies, hygienic conditions, and staff with technical capacity. In contrast, supply methods such as oral contraceptives and condoms are typically obtained from private pharmacies and mobile units [15].

Contraceptive use is associated with sociodemographic characteristics of the user and with the supply source of the method [15-19]. The use of modern contraceptives among women who obtained their method from public sources tends to be positively associated with number of living children. Educated and wealthier women are more likely to be able to pay for their contraception and they may get it from the private sector, while poor or uneducated women may rely more on the public sector for their methods. Women living in urban areas may have greater access to a wide range of contraceptive services and methods as compared to rural women.

Since 1988, the IUD has remained the most popular contraceptive method in Egypt [20]. Before that, oral contraceptive was the leading method used by Egyptian women. The prevalence of IUD use among married women increased from 4% in 1980 to 37% in 2003 [20]. Because it is a clinical method and the most common method of contraception, IUD use is a good proxy indicator to measure clinical contraceptive use in Egypt. This study examines the association between IUD use among married women age 15–49 and the quality in the provision of family planning services, adjusting for several factors known to be associated with contraceptive use.

## Methods

The analysis is based on the 2003 Egypt Interim Demographic and Health Survey (EIDHS) [20], and the 2002 Egypt Service Provision Assessment (ESPA) survey [21]. The EIDHS collected information from 921 clusters. A total of 10,089 sample households were selected, including 4,611 in urban areas and 5,478 in rural areas. Overall, 9,217 women age 15–49 in the sample households were eligible for interview. Individual questionnaires were completed for 9,159 (99%) of eligible women, 8,445 of whom were currently married. More details on the EIDHS sample design are provided in the main survey report [20]. The ESPA collected information from a sample of 650 health facilities, including 602 facilities offering family planning services. Latitude and longitude coordinates were collected using global positioning system units for the center points of the communities where the EIDHS survey respondents lived, and for the ESPA surveyed health facilities.

The EIDHS data were linked with the ESPA facility data using geographic information system (GIS) methods. Using the latitude and longitude coordinates, distances were calculated between the EIDHS communities and the ESPA family planning facilities. Women in an EIDHS cluster located within 10 km from a surveyed family planning facility were included in the analysis. Among the 602 facilities offering family planning services, 585 were within 10 km from a EIDHS cluster center. For the EIDHS, 854 clusters were linked to at least one ESPA facility within a distance of 10 km from the cluster center, and 766 of these clusters were linked to at least one facility offering family planning services. Women from EIDHS clusters that did not link to any ESPA facility offering family planning services within their 10 km range were excluded from the study. If a cluster was linked to more than one family planning facility, the one that was closest to the center of the cluster was included in the analysis. The linkage generated an EIDHS-ESPA matched sample of women and family planning facilities, including 6,735 married women or 80% of the original EIDHS sample.

The outcome variable of this study is the prevalence of current IUD use among married women age 15–49. The outcome variable was divided into four categories: IUD obtained from public facilities, IUD obtained from private facilities, other contraceptive methods, and no contraceptive use.

The main predictor variable is the index of quality of family planning services, which is based on the availability of 22 items that support the provision of family planning services. Items are grouped into four dimensions of quality of family planning services: 1. *counseling*, including family planning guidelines, client privacy, visual aids for family planning, and individual client card; 2. *examination*, including availability of private room, examination bed/table, spotlight, speculum, soap, water, latex gloves, disinfecting solution, and sharp box; 3. *supply*, including availability of oral contraceptives, injectables, condoms, norplant, IUD, and rhythm method; and 4. *management*, including whether the facility has 50% or more providers who received training in the last 12 months, whether 50% or more providers were supervised in the last 6 months, and whether the family planning register had been updated in the last 7 days. These four dimensions of quality measure infrastructure and system readiness of a facility.

The quality indices for each quality dimension of family planning services are the total availability of items in each dimension standardized to add to 25. The total family planning quality index is the sum of all 4 component scores and has the maximum value of 100 (Table 1). The differences in quality of family planning services are measured by dividing the total scores into three quintiles representing the low, medium, and high quality of family planning services. The analysis is conducted after controlling for a number of factors that are known to be associated with the use of modern contraceptives, including distance from home (cluster) to the nearby surveyed family planning facility (< 4 km, ≥ 4 km), type of family planning facility (government hospital, MCH/urban health unit, rural health unit, mobile clinic/health office, NGO), age of woman (15–19, 20–24, 25–29, 30–34, 35–39, 40–44, 45–49), number of living children (0–2, 3+), education level (no education, some primary, primary complete, secondary or higher), household wealth status (lowest, second, middle, fourth, highest), and residence (urban, rural).

Effects of the quality of family planning services and other factors on the use of IUD and other contraceptive methods were estimated using multinomial logistic regression procedure in Stata 8.0 [22]. In the survey, certain categories of the respondents were over-sampled and non-response rates varied from one geographical area to another. In our analysis, weights were used to restore the representativeness of the sample. Results are presented as relative risk ratios (RRR) with statistical significance levels (*p*-values). Estimation of standard errors takes into account design effects due to clustering at the level of the primary sampling unit.

## Results

The distributions of the total sample and matched sample by variables included in the analysis are very comparable. In the matched sample, about 37% of currently married women presently used IUD, 24% got their IUD from a public source and 13% from a private source; while 20% used other contraceptive methods and 43% did not use contraception. Fifty-six percent of women lived within 4 km from a sampled family planning facility. Age distribution shows that 4% of women were aged 15-19, 16% were 20-24, 21% were 25-29, 16% were 30-34, 17% were 35-39, 14% were 40-44, and 12% were 45-49. Fifty-four percent of women had three or more living children. Slightly more than one-third had no education, 13% had some primary education, 14% had completed primary education, and the remaining 37% had completed secondary or higher education. By household wealth status, 18% and 19% of the women live in the lowest and the second quintiles, respectively, and 21% each live in the middle, fourth, and highest quintiles, respectively. Forty-two percent of the women lived in urban areas and 58% lived in rural areas.

### Unadjusted effects of services quality and other factors on the use of IUD

The unadjusted relative risk ratios show that the quality of family planning services has a significant positive effect on the use of IUDs from public sources (RRR = 1.23; *p *< 0.05). The use of IUD obtained from a private source and the use of other contraceptive methods are not associated with the quality of family planning service. The distance from home to a surveyed health facility is strongly negatively associated with the use of IUD at both public (RRR = 0.86; *p *< 0.05) and private sources (RRR = 0.54; *p *< 0.01), but not with the use of other contraceptive methods. Use of IUDs and other methods is not associated with the type of facility, except for mobile unit/health office. The reverse U-shape of the unadjusted effects of age shows a strong significant relationship between age and use of IUD from both sources as well as for other contraceptive methods. As expected, having 3 or more living children is significantly positively associated with the use of any contraceptive method. The positive unadjusted effect of education level is significant among IUD users from private sources. Household wealth status has monotonic strong positive effect on the use of IUD from private sources. Its effects on the use of IUD from public sources fluctuates somewhat, and it is not associated with the use of other contraceptive methods. Rural residence is significantly negatively associated with the use of IUD from public sources (RRR = 0.80; *p *< 0.05), from private sources (RRR = 0.43, *p *< 0.01), and other contraceptive methods (RRR = 0.86; *p *< 0.10).

### Adjusted effects of services quality and other factors on the use of IUD

In the multivariate model, the effects of family planning services quality on the use of IUD obtained from public sources is further sharpened when the analysis is adjusted for distance to the facility, type of facility, age, education level, number of living children, household wealth status, and residential area (RRR = 1.36, *p *< 0.01). Among the control variables, woman's age and her number of living children remain significantly associated with the use of IUD and other contraceptive methods. Having secondary or higher education is significantly positively associated with the use of IUD obtained from private facilities. The adjusted effect of household wealth status is monotonically positively associated with the use of IUD obtained from private sources. However, the use of IUD from public sources is positively associated with only the second, middle, and fourth quintiles of household wealth status. Rural residents remain negatively associated with the use of IUD from public sources and use of other contraceptive methods.

Table 4 presents the unadjusted and adjusted effects of four separate dimensions of quality of family planning services on the likelihood of IUD use and other methods. The results show that the effects of total quality in family planning services on the use of IUD obtained from public sources is contributed by two main quality components: counseling and examination, independent of several other factors.

## Discussion

Egypt has been pursuing a population control program by stimulate demand for family planning and promote use of contraceptive services through better supply, training and supervision of providers [23]. Results from this study show that quality of family planning services has a significant positive effect on the use of IUD obtained from public sources, independent of distance to the facility, facility type, age, number of living children, educational level, household wealth status, and residence. A similar effect is not observed for the use of IUD from private sources, perhaps mainly because the ESPA sample only included public facilities and private not-for-profit facilities. The use of other methods is not associated with the quality of family planning services in the nearly facility, perhaps because these methods are less likely to be obtained in the facilities. Results from the analysis of four separate quality dimensions suggest that, in Egypt, IUD use is independent of the supply of contraceptive methods as previously observed [24] and is driven mainly by quality of counseling and examination room services.

A reverse U-shaped relationship between woman's age and use of IUD, as well as other contraceptive methods, is consistent with findings from other developing countries [15]. For women of reproductive age, the benefits of using contraceptives are greater than the associated risks and side effects. The positive relationship between number of living children and IUD use has also been demonstrated elsewhere [25]. In previous studies, economic status has been identified as one of the key factors affecting contraceptive use. The results from this study show that in Egypt household wealth status is associated with IUD use from private sources, perhaps because services from public or not-for-profit private sources can be obtained with minimal cost, whereas access to private-for-profit services usually involves higher cost. However, studies from Vietnam and Lao PDR have shown that socioeconomic status is not a determinant of utilization of private health services [26,27].

One of the major constraints of this study is that it links each individual woman to the nearest facility that was surveyed for the ESPA, and these facilities may or may not include the facility where an individual woman may have received her IUD or other contraceptive method. The ESPA included only a sample of facilities, not the census of all facilities in the country. The linked facility data used in this study do not allow examining quality of care in the facility actually visited and obtained contraceptive methods. Therefore the analysis does not have sufficient information to adjust for the preferred facility or the facility actually visited. This is particularly so in urban settings. However, in rural areas where the density of facilities is much lower, it is likely that most women were linked to the facilities that they actually used for obtaining family planning.

Another potential constraint is that life span of the IUD itself extends from several months to a few years, and women who currently use an IUD may have acquired their method recently or sometime ago. The results presented here would better reflect the association if the analysis included only women who recently adopted IUD. However, the median duration of IUD use in EIDHS sample was 17 months, and the changes in the quality of health services affected by changes in the facility structure, services, supplies, and management are latent progressive processes and would not differ markedly within a one- or two-year period [21,28].

## Conclusion

This study is one among a few that uses geographic information to link data from a population-based survey with an independently-sampled national health facility survey to analyze the association between quality of family planning services and use of IUD [29,30]. Results in this study are similar to those from an earlier study in 15 developing countries, which also showed that quality is an important determinant of contraceptive use [31]. Findings from a study in Morocco also indicated that distance to a public health center was associated with higher utilization of a modern contraceptive method and lower discontinuation rates; while the presence of a private source for contraceptive methods, a pharmacy, was associated with higher discontinuation rate [32]. The findings demonstrate that service quality is an important determinant of use of clinical contraceptive methods in Egypt. Improving quality of family planning services may help further increase use of clinical contraceptive methods and reduce fertility.

## Competing interests

The author(s) declare that they have no competing interests.

## Authors' contributions

Rathavuth Hong carried out the study design, data management and analysis, and drafted the manuscript. Livia Montana carried out the GIS analysis and revised the manuscript. Vinod Mishra participated in the study design and revised the manuscript. All authors read and approved the final manuscript.

## Pre-publication history

The pre-publication history for this paper can be accessed here:



## Figures and Tables

**Table 1:** Quality dimensions and items in the quality index of family planning services, Egypt SPA 2002

**Quality dimension and item**	**Definition of item**	**Score**
*1. Counseling*		*25*
Guideline	Guidelines or protocols on counseling	25/4
Privacy in counseling room	Private room that ensures visual and auditory privacy	25/4
Visual aids	For demonstrating the use of family planning methods	25/4
Individual client card	Individual client card or record or chart for family planning	25/4
		
*2. Examination room*		*25*
Privacy in examination room	Private room that ensures visual and auditory privacy	25/9
Examination table/bed	Table or bed for examination such as pelvic examination	25/9
Source of light	Examination light including spot light	25/9
Speculum	Vaginal speculum for pelvic examination	25/9
Soap	For hand washing	25/9
Water	For had washing (tap water or bucket with tap)	25/9
Glove	Clean (disposable or sterile) latex gloves	25/9
Decontamination solution	Mixed solution for hand decontamination	25/9
Sharp box	For disposing used sharp objects (i.e. used needles)	25/9
		
*3. Supply of contraceptive methods*		*25*
Oral contraceptives	Combined or progesterone-only pills	25/6
Injectables	Combined or progesterone-only injection	25/6
Condoms	Male condoms	25/6
Implants	Norplant or Implanon	25/6
IUDs	Intrauterine devices	25/6
Rhythm	Counseling on natural or rhythm method	25/6
		
*4. Management*		*25*
≥ 50% of staff received training	In-service training in any family planning topic in past year	25/3
≥ 50% of staff received supervision	Receiving personal supervision in past 6 months	25/3
Having up-to-date registration	Having a recorded register up-to-date in last 7 days	25/3
		
Total score		100

**Table 2:** Distribution of the sample of married women age 15–49 in the original sample and in the matched sample by selected characteristics, Egypt SPA 2002; Egypt IDHS 2003

**Background characteristic**	**Total sample**		**Matched sample**	
		
	N	Percent	N	Percent
Status of contraceptive use				
Use IUD, public sources	2035	24.1	1,645	24.4
Use IUD, private sources	1060	12.6	862	12.8
Use other methods	1685	20.0	1,327	19.7
Not use contraceptive	3664	43.4	2,901	43.1
Quality of family planning index				
Low	-	-	2,171	32.2
Middle	-	-	1,998	29.7
High	-	-	2,566	38.1
Distance to the nearest facility				
< 4 km	-	-	3,774	56.0
≥ 4 km	-	-	2,961	44.0
Type of facility				
Government hospital	-	-	815	12.1
MCH/Urban health unit	-	-	938	13.9
Rural health unit	-	-	2,134	31.7
Mobile/Health office	-	-	1,342	19.9
NGO	-	-	1,506	22.4
Age				
15–19	332	3.9	274	4.1
20–24	1,343	15.9	1,080	16.0
25–29	1,703	20.2	1,383	20.5
30–34	1,346	15.9	1,056	15.7
35–39	1,462	17.3	1,156	17.2
40–44	1,205	14.3	954	14.2
45–49	1,054	12.5	832	12.4
Number of living children				
0–2	3,820	45.2	3,080	45.7
3+	4,625	54.8	3,655	54.3
Education				
No education	3,080	36.5	2,409	35.8
Some primary	1,053	12.5	869	12.9
Primary complete	1,190	14.1	936	13.9
Secondary/higher	3,122	37.0	2,522	37.4
Wealth status^1^				
Lowest	1,525	18.1	1,187	17.6
Second	1,621	19.2	1,283	19.0
Middle	1,742	20.6	1,420	21.1
Fourth	1,793	21.2	1,401	20.8
Highest	1,765	20.9	1,444	21.4
Residence				
Urban	3,589	42.5	2,859	42.5
Rural	4,856	57.5	3,876	57.6
				
Total	8,445	100.0	6,735	100.0

There are 7,367 married women from clusters that match with a facility within 10 km from cluster's center, but only 6,735 of them match with a facility that offer family planning services

^1^The Wealth Index measures a household's relative economic status based largely on household ownership of durable assets. For details on the calculation of the index, see [20].

**Table 3:** Effects of the quality in family planning services and other selected factors on the use of IUD among married women age 15–49, Egypt SPA 2002; Egypt IDHS 2003

**Characteristic**	**Unadjusted RRR**			**Adjusted RRR**		
		
	Public	Private	Other methods	Public	Private	Other methods
Quality index						
Low^a^	-	-	-	-	-	-
Middle	1.12	0.86	1.02	1.20*	0.86	1.08
High	1.23**	1.02	1.01	1.36***	1.05	1.09
Distance to nearest facility						
< 4 km^a^	-	-	-	-	-	-
≥ 4 km	0.86**	0.54***	1.01	0.97	0.92	1.08
Type of facility						
Hospitals^a^	-	-	-	-	-	-
MCH/Urban health unit	1.04	1.22	1.10	0.87	0.74	0.94
Rural health unit	0.89	0.78	0.91	0.82	0.76	0.84
Mobile/Health office	1.06	1.48**	0.79	0.98	0.94	0.75*
NGO	1.09	1.13	0.99	1.07	0.82	0.93
Age						
15–19^a^	-	-	-	-	-	-
20–24	2.54***	3.61***	2.83***	2.36***	2.41**	2.41***
25–29	3.59***	5.19***	3.54***	2.56***	2.64**	1.98***
30–34	5.45***	10.63***	6.18***	3.17***	4.10***	2.46***
35–39	6.90***	11.47***	8.11***	3.63***	4.26***	2.78***
40–44	5.64***	11.28***	7.33***	2.70***	3.93***	2.30***
45–49	1.71**	3.66***	2.94***	0.79	1.24	0.88
Number of living children						
0–2^a^	-	-	-	-	-	-
3+	2.18***	1.95***	3.38***	2.43***	2.66***	3.94***
Education						
No education^a^	-	-	-	-	-	-
Some primary	1.06	1.30	0.92	1.02	1.10	0.94
Primary complete	1.06	1.39**	0.92	1.15	1.21	1.19
Secondary/higher	0.97	2.91***	0.83	1.06	1.96****	1.16
Wealth status						
Lowest^a^	-	-	-	-	-	-
Second	1.41***	1.98***	1.03	1.47***	1.90***	1.10
Middle	1.35***	2.31***	0.94	1.44***	2.17***	1.04
Fourth	1.58***	3.35***	0.98	1.50***	2.70***	1.01
Highest	1.26*	7.82***	1.02	1.19	5.32***	1.08
Residence						
Urban^a^	-	-	-	-	-	-
Rural	0.80***	0.43***	0.86*	0.80**	0.93	0.75**
						
Number of married women	6,645			6,645		

^a ^Reference group

*p < 0.1, **p < 0.05, ***p < 0.01

**Table 4:** Unadjusted and adjusted effects of individual components of quality in family planning services on the use of IUD among married women age 15–49, Egypt SPA 2002; Egypt IDHS 2003

**Component of quality**	**Unadjusted RRR**			**Adjusted RRR**^b^		
		
	Public	Private	Other methods	Public	Private	Other methods
Counseling						
Low^a^	-	-	-	-	-	-
Middle	1.22**	0.83	0.97	1.39***	0.94	1.08
High	1.36***	1.00	1.02	1.61***	1.13	1.15

Examination room						
Low^a^	-	-	-	-	-	-
Middle	1.16*	1.17	0.93	1.14	1.09	0.91
High	1.49***	1.14	1.06	1.46***	1.02	1.04

Supply of contraceptive						
Low^a^	-	-	-	-	-	-
Middle	1.08	0.98	1.02	1.09	0.94	1.01
High	0.89	0.89	1.20	0.78	0.78	1.04

Management						
Low^a^	-	-	-	-	-	-
Middle	1.11	0.99	1.15	1.24*	1.01	1.34**
High	0.90	0.89	1.04	1.03	0.94	1.25

^a ^Reference group

^b ^Adjusted RRR based on the full model but only main effects are presented

*p < 0.1, **p < 0.05, ***p < 0.01
